# Horses as a Crucial Part of One Health

**DOI:** 10.3390/vetsci7010028

**Published:** 2020-02-29

**Authors:** Nelly Sophie Lönker, Kim Fechner, Ahmed Abd El Wahed

**Affiliations:** Virology Lab, Division of Microbiology and Animal Hygiene, University of Goettingen, Burckhardtweg 2, 37077 Goettingen, Germany; n.loenker@stud.uni-goettingen.de (N.S.L.); kfechne@gwdg.de (K.F.)

**Keywords:** One Health, horse-human-ecosystems, risk factor interactions, zoonosis, noncommunicable diseases (NCDs)

## Abstract

One Health (OH) is a crucial concept, where the interference between humans, animals and the environment matters. This review article focusses on the role of horses in maintaining the health of humans and the environment. Horses’ impact on environmental health includes their influence on soil and the biodiversity of animal and plant species. Nevertheless, the effect of horses is not usually linear and several factors like plant–animal coevolutionary history, climate and animal density play significant roles. The long history of the relationship between horses and humans is shaped by the service of horses in wars or even in mines. Moreover, horses were essential in developing the first antidote to cure diphtheria. Nowadays, horses do have an influential role in animal assisted therapy, in supporting livelihoods in low income countries and as a leisure partner. Horses are of relevance in the spillover of zoonotic and emerging diseases from wildlife to human (e.g., *Hendra Virus*), and in non-communicable diseases (e.g., *post-traumatic osteoarthritis* in horses and *back pain* in horse riders). Furthermore, many risk factors—such as climate change and antimicrobial resistance—threaten the health of both horses and humans. Finally, the horse is a valuable factor in sustaining the health of humans and the environment, and must be incorporated in any roadmap to achieve OH.

## 1. Introduction

One Health (OH) is a holistic approach which defines the health of humans, animals and the environment as a coherent system. OH as defined by the WHO includes the design and deployment of policies, legislation and research at multidisciplinary level to assure better public health [1]. Hippocrates (460 BC–370 BC), the Greek physician and “father of modern medicine”, mentioned in his text entitled “On airs, waters and places” (400 BC) that the health of humans depends on a clean environment. He emphasized the importance of medical researchers incorporating geographical locations, climate conditions and the quality of drinking water as factors affecting health and susceptibility to diseases. The interdisciplinary approach of OH combines veterinary and human medicine, environmental science, wildlife biology and public health. OH becomes increasingly important as 61% of infectious agents are zoonotic [2]. Several factors influence OH as increasing travel behavior, global trade and change in land use, population growth of humans and animals as well as an increasing use of animal products [3,4]. The idea of OH can be traced back to ancient times, when traditional healers treated both animals and humans. Moreover, plant and animal components (e.g., hooves and bones) were used to cure diseases in humans [5]. Nowadays, comparative medicine, an interdisciplinary field of veterinary and human medicine, integrates animals as an important factor in unearthing the origin of diseases. Therefore, studying the human/animal interface is crucial. 

**Figure 1:**
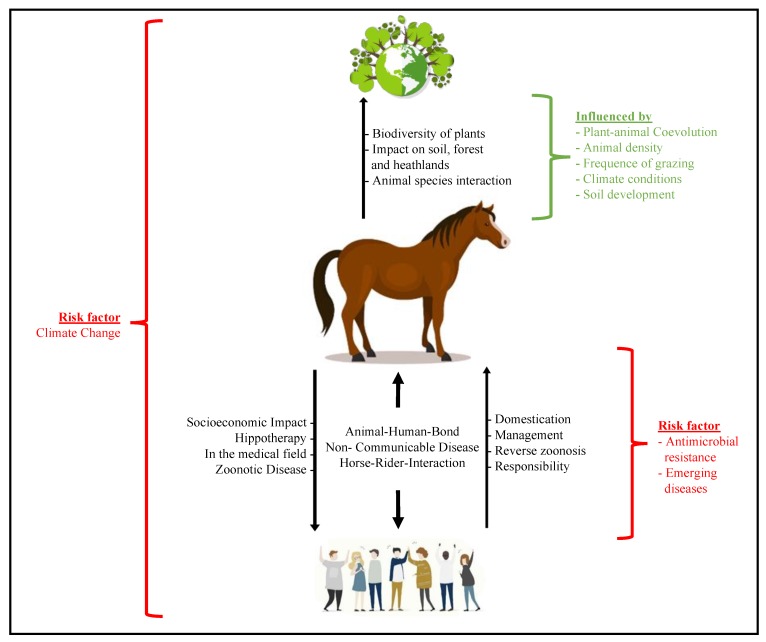
Horse, human and environment interaction pathways. (Symbols: freepic.com).

Horses are among the most important animals in human history; they have been used in wars, as a means of transport, and even facilitated work in mines. In the late 19th century, horses played a crucial part in developing the first antidote to cure diphtheria. Since then, the rate of contact between domesticated horses and humans steadily increased. Nowadays, for example, horses play an important role in animal-assisted therapy. Furthermore, the detection of infectious diseases that affect both humans and horses are crucial, especially in cases of highly transmissible diseases. Beside infectious diseases, non-communicable diseases (NCDs) such as skeletal and joint diseases or metabolic disorders are of concern to both. Several risk factors concerning the health of humans and horses exist. Therefore, this article will give an overview of OH with a focus on horses and their relation to the environment and humans, along with the influence of other factors such as climate change (see Figure 1).

## 2. The Horse-environment Relationship

Feral horses (*Equus caballus*) can be found on every continent of the earth except Antarctica [6], and have an impact on soil and vegetation [7,8], the biodiversity of plants [9], and several animal species, such as reptiles and small mammals [10,11], ants [7], herbivores [12,13] and grassland birds [14]. Nevertheless, among the genus *Equus* itself, great diversity can be observed [15]. From an OH perspective, horses’ influence on the environment depends strongly on several factors such as plant-animal coevolutionary history, soil development, climate, frequency of grazing, and animal density [10]. Horses play an important role in increasing plant diversity through acting as natural fertilizer and by the dispersal of plant species [9,16]. Accordingly, horses can positively influence the biodiversity of both plants and animals. In southwestern Spain, free-ranging Galician mountain ponies can help in preventing forest fires by eating small plants growing under the shades of long trees. Moreover, the consumption of scrub has a positive influence on the maintenance of threatened heathlands and the variety of species living there. Furthermore, those ponies are the most important source of food for wolves; consequently, this leads to the indirect protection of farmers´ livestock, since wolves do not hunt their animals [17]. The grazing habits of the wild horse have a significant impact on other animal species; for instance, horses consuming *Spartina* grass destroy the nesting habitats of laughing gulls (*Leucophaeus atricilla*)—however, as a result, the shore bird diversity will increase [18]. The presence of wild horses leads to a significant reduction of the desert bighorn sheep and elks sharing the same water source, which is evidence of indirect competition [12,13]. Wild horses can have a negative impact on environmental health, for example horses’ trampling leads to an increased soil strength and compaction, which reduces rainwater infiltration [8]. This in return can lead to nutrient and water shortages for plants and trees. Horses´ bark chewing behavior increases the mortality of trees, which results in changes in vegetation composition and structure [19,20]. These examples clearly show the influence of the horse is not linear, but rather depends upon the surroundings. Thus, a negative influence on one species can have a positive effect on another.

## 3. The Domestic Horse-human Relationship

In contrast to wild horses, domesticated horses live in close contact with humans. The research of Warmuth et al. (2012) discovered that the domestication of horses started approximately 6000 years ago in the Ukraine, southwest Russia and west Kazakhstan [21]. The domestication of animals can be a result of “humans’ innate tendency to focus on life and lifelike processes”, referred to as *Biophilia hypothesis* by Edward O. Wilson [22]. To demonstrate the strong animal–human bond, a human´s loss of a companion animal—whether natural or by euthanasia—is associated with deep grief and depression [23]; however, compared to other pet animals, horses are frequently sold when not “suitable” anymore [24]. Furthermore, the use of horses strongly depends on the cultural background. In some countries, horses are needed for work, and thus have an impact on the economic status of the owner. In high-income countries, horses are primarily used for sport, breeding, animal assisted therapy, or as companions for leisure. Besides that, horse meat is a common food source, especially in France, Mexico and Argentina [25]. Despite animals (e.g., cattle in Hinduism) playing an important role in religion [26], which directly affects human mental health [27], horses are not part of any religion. Nevertheless, horses have always been deployed as a sign of power. During the Iron Age, a horse would be sacrificed during the burial of its leader [28]. In Roman times, white horses were considered to be holy [29]. Horses were also associated with a goddess, Epona, who represented horsemanship [30].

### 3.1. Horse-rider Interactions

Recently, studies on horseback riding and the horse-rider interaction (HRI) have been crucial in assuring horse welfare. HRI factors include harmony, coordination dynamics, motor coordination, phase synchronization, and periodicity [31]. The rider is mainly responsible for keeping the horse healthy, trained and motivated, since many horses (e.g., in the UK: 40% of 11,363 dressage horses) do suffer from back pain due to equestrian sport [32,33]. On other hand, HRI is also important for the riders, as they often suffer from back pain as well [34]. Besides physiological aspects of HRI, the psychological interactions between horses and rider are important, but not always easy to follow, since horses are unable to verbalize their pain and emotions [32], whilst still being social animals with highly sensitive reactions to the rider [35]. Gathering information on how they influence one another is important to provide improved training methods for horses and riders to assure health benefits and the maintained physical fitness of both. 

### 3.2. Equine Assisted Therapy

In hippotherapy, horse movements are used to support the human healing process of skeletal and neurological disorders like infantile cerebral palsy [36,37] and multiple sclerosis [38,39]. Several studies have also reported about the positive influence of hippotherapy on people affected by Down’s syndrome [40], autism spectrum disorder [41], or on post-stroke patients [42]. During horse riding, the pelvis of the rider moves in a smooth, rhythmic, and repetitive pattern [43]. Impaired people have the chance to adapt their motion apparatus to the movement and rhythm of the horse [37]. Consequently, the patient´s regulation of the muscle cycle, breathing rhythm, strength of the torso muscle, improvement of balance, coordination and symmetry is positively influenced [39,44]. Therefore, the proper position of the rider on the horse’s back is necessary to reach optimal physical health benefits [45] and to disturb the horse’s back and movement as little as possible. Beside the physical impact, the proximity to horses and therapists positively affects the emotional, social and mental well-being of the patients [42], for instance, therapeutic horseback riding is used to support the emotional status of humans with autism spectrum disorder [46]. Moreover, handling patients in nature can have a positive influence on the quality of life of patients compared to routine therapeutic settings in hospitals [45]. However, many obstacles might prevent hippotherapy, such as patients being afraid of horses or financial barriers [43].

### 3.3. Socioeconomic Impact of Working Horses

The working horse can have a drastic impact on the socioeconomic status of the owner and consequently on mental health particularly in case of disease or death [47]. In low income communities, the owner´s livelihood is limited by factors such as poverty, low status and limited access to resources. Therefore, working horses enhance capital and secure sustainable livelihoods [48]. In central Ethiopia, where horses are used as cart taxis, the loss of working horses restricted business and signified a major economic crisis for the local community [49]. Chang et al. (2010) found out that low-income communities have achieved a positive income using working horses [50]. Besides the economic factors, owning working horses can benefit status, leading to stronger social relations [51]. However, national policies and institutions that monitor and support working with horses are often weak or non-existent in developing countries [52]. Consequently, illness and injuries of working horses are often the result of a lack of knowledge of wound and disease management. Other reasons that influence the performance of working horses are overloading the horse, insufficient access to water and food as well as veterinary care and inadequate recovery phases [53].

## 4. Horses in the Medical Field

In the 1890, horses played a crucial part in developing the first antidote to cure diphtheria (*Corynebacterium diphteriae*) in humans [54]. Horse serum is also used as an anti-venom, for example, when humans are bitten by snakes (e.g., Brazilian Bothrops or Crotales) [55]. However, horse serum can cause an allergic reaction in humans named serum sickness, as reported after the introduction of the diphtheria-antitoxin in 1894 and with equine rabies immunoglobulin [56,57]. Wilde et al. (1989) point out the importance of standardizing the purification methods and potency criteria to achieve as low side effects as possible [58]. 

As an animal model, horses were used in the research of *Hepatitis C*, since the virus displays great similarities to the *equine hepacivirus* [59,60]. Furthermore, horses were employed as a model for respiratory diseases (*human allergic neutrophilic asthma*) [61], orthopedic problems (focal articular cartilage injuries) [62,63], and in defining the causes of depression as an ethological animal model [64].

## 5. Zoonotic Diseases

Several infectious diseases are known to affect both horses and humans, see Table 1. Zoonotic diseases are those transmitted from horse to human through direct contact (e.g., *Hendra virus*) or indirect infections due to vehicles such as food products (e.g., *Botulism*) or vectors like ticks (e.g., *Lyme-Borrelioses*) and mosquitos (e.g., *West Nile Fever*) [65]. Zoonotic diseases are mostly an interface between wildlife, animals and humans [66]. Therefore, the transfer rate of zoonotic diseases highly depends on the immune status of the animals and humans, pathogen-control measurements and the contact to wildlife [67]. To monitor the transmission of zoonotic infections, several surveillance systems do exist [66]. Despite many reports on zoonotic diseases, no study has been recorded on reverse zoonosis, where human is the main source of infection to horses. 

## 6. Non-communicable Diseases

Besides infectious diseases, non-communicable diseases (NCDs) such as skeletal and joint diseases, cardiovascular problems, psychological problems or metabolic disorders are of great concern for horses and humans. NCDs are the result of genetic, physiological, environmental and behavioral factors and persist over a long duration of time [103]. Since horses are often kept in stables, physical inactivity may be a man-made reason for skeletal and joint diseases in horses. On other hand, the extensive training of a horse can lead to NCDs, e.g., post-traumatic osteoarthritis [104] and back problems [32]. Psychological disorders can evolve because of inappropriate housing management, weaning methods, and social or feeding restrictions [105,106,107]. Horses can develop stereotypic behavior—such as weaving, wind sucking, crib biting or box walking—when living in a poor and unattractive environment [105,108]. Furthermore, unhealthy diets, especially highly concentrated and sugar-containing diets, can cause diseases such as equine metabolic syndrome [109]. If keeping horses in stables with bad air quality and low hygiene, horses and their keepers can develop respiratory problems, like the chronic obstructive pulmonary disease [110,111]. Other NCDs such as Colitis-X, Caprophagy in foals or Buttress foot can be influenced by humans as well [112]. Since the welfare of farm animals is the human responsibility, several welfare concepts, like “The five freedoms”, exist to prevent management-related diseases [113]. Pain in the lower back, hip joint and hamstring muscles are the most common horse riders’ orthopedic problems [114].

## 7. Risk Factors

There are various risk factors, such as climate change and antimicrobial resistance, which have a negative impact on the health of both humans and horses. In order to counter this, the U.N. developed the “Sustainable Development Goals” focusing on 17 targets, such as “Zero Hunger”, “Good Health and Well-Being” and “Climate Action” [115], with the ambition to reach a sustainable future for everyone. Even those goals are anthropocentric, from a OH perspective horses are a crucial part in Goal 3 “Good Health and Well-Being”, since this objective aims to reduce the spread of infectious diseases. As already mentioned above, the health of working horses is significant for the income in certain communities. Therefore, horses are also of relevance to Goal 2 “Zero Hunger”.

### 7.1. Climate Change

The global earth warming has among others an impact on food security and health issues [116]. Climate change affects the availability of freshwater resources, natural ecosystems and agriculture [117]. The food crisis in 2006–2008 caused by the rising demand for food and animal feed products combined with factors such as drought periods and crop diseases, demonstrated the insecurity of our ability to feed the world [118]. Toreti et al. (2019) point out the need to improve adaption strategies in agriculture management to defend weather-related crop diseases and plant pests [119]. Rising temperatures also affect the spread of infectious diseases, e.g., West Nile Virus recorded in new niches as consequence of changes in the mosquito’s lifecycle and distribution range [116]. Likewise, the migration behavior of wild birds (amplifying hosts of, e.g., West Nile Virus and Borrelia burgdorferi) is changing, which leads to the emergence of new infection centers [120,121,122,123]. De la Roque et al. (2008) stressed that extreme weather conditions are of greater concern in the transmission of infectious diseases [117]. For instance, heavy precipitations are associated with a higher number of Hanta virus infections, where the horse farm environment is an excellent site for rodent breeding. Therefore, horse owners or care takers are more likely to be infected with Hanta virus [124]. The latter is an example of the indirect involvement of horses in the spread of diseases. NCDs are affected with by weather conditions as, e.g., cardiovascular problems and respiratory diseases are associated with rising heat waves and environmental pollution [125,126]. Hence, stable management and training should be adapted in order to prevent heat stress. Nevertheless, to protect public and animal health from the adverse effects of climate change, it is necessary to introduce vaccination campaigns, public education, suitable technologies such as water filtration and insecticides, and surveillance systems such as weather warnings and disease monitoring with a focus on the migration behavior of animal species.

### 7.2. Antimicrobial Resistance

In 1951, Starr and Reynolds published the first report on suspected antimicrobial resistance (AMR) in livestock farming [127]. Since then, AMR are rapidly rising due to factors like global trade and the intense international transport of animals [128]. Harbarth et al. (2015) refer to the fact that the extensive use of antibiotics always stressed bacteria to develop mutations, or recombinant pieces of DNA to resist antibiotic [129]. The use of growth-promoting antibiotics in industrial livestock farming is a trigger for the AMR [130]. From the OH-perspective, the same antibiotic is deployed in veterinary and human medicine [131]. Consequently, the transfer of resistant strains to humans can occur through food products, direct contact or contaminated environments [128]. AMR in horses was first documented in 1970 [132], almost twenty years after the first discovery in animals. Several studies report about methicillin-resistant staphylococcus aureus (MRSA) and extended spectrum beta-lactamase (ESBL)-producing Enterobacteriaceae [89,133,134], which also risks human’s health [92]. Van Duijkeren et al. (2011) identified the possible transmission of MRSA from a horse to a human [135]. Recently, Salmonella typhimurium cases have raised the awareness of multidrug resistance in horses [136,137]. Finally, the responsibility for the administration of antibiotics to animals lies in the hands of humans; therefore, strict guidelines must be implemented to achieve OH.

### 7.3. Emerging Diseases 

Both novel infectious diseases or new phenotypes of known pathogens have a huge impact on horse/human health and relationships. Important emerging diseases are depicted with an “E” superscript in Table 1. The most prominent example of emerging disease is Hendra virus, which was implicated in fatal cases in both humans and horses in Australia. The virus belongs to the *paramyxoviridae* genus, with a natural reservoir in flying foxes. Hendra virus is directly transmitted when people are in contact with the secretions of an infected horse [92]. Middleton et al. (2014) discussed the Hendra virus vaccine for horses as a OH approach, since it protects horses, human and the environmental health as the eradication of infected flying foxes is not an option because of the massive impact it would have on the ecosystem [71]. Recently, the epidemic of coronavirus disease 2019 (COVID-19) triggers the question of a possible animal reservoir. Around 10% of horses in the USA were positive for β-coronavirus [138], which is the cause of the COVID-19. No direct relation between the human and horse coronaviruses was recorded, but the coronavirus is a highly mutated microorganism, which could always jump the species barrier [139].

## 8. Conclusions

Despite the need of OH several decades ago, only recently has more attention been given. Discussions at the scientific level are urgently needed; however, the involvement of the public and community members at local, national and global levels is essential to assure the implementation of OH. Cooperation between “open-minded” interdisciplinary professions bringing together OH aspects from various research areas will be a strong approach to increasing awareness beyond the anthropocentric perspective. In other words, keeping animals and environment health primarily for the service of human health is no longer valid. If a `radical` OH approach assumes that the health of humans, animals and the environment are of equal value to one another and interactive, then the responsibility of humans becomes immediately apparent, especially in the case of man-made issues. The public must be educated before direct interaction with horses; therefore, legislation with social license to operate the horse is necessary. While the OH approach is often associated with zoonotic infections, other OH aspects are important, especially the NCDs and maintaining a healthy environment as well as the production of effective vaccines. Ultimately, the horse as a crucial part of OH represents a valuable opportunity to demonstrate the constructive interference of OH aspects between animals, the environment and humans.

## Figures and Tables

**Table 1:** Horse/human zoonotic and emerging diseases. Z is zoonotic and E is emerging.

				Transmission			
				Direct	Indirect		
Disease	Pathogen	Family	Reservoir		Vector	Vehicle	Reference
**Viruses**							
Borna ^Z^	*Borna Disease Virus-1*	Bornaviridae	Shrew species (*Crocidura leucodon*)	Olfactory route			[68,69]
Eastern Equine Encephalitis Virus ^Z,E^	*Alphavirus*	Togaviridae	Wild birds and rodents		*Aedes, Culiseta, Culex*		[65,70]
Hendra ^Z,E^	*Hendravirus*	Paramyxoviridae	flying fox (*Pteropus bats*)	Nasal discharge			[71]
Rabies ^Z^	*Lyssavirus*	Rhabdoviridae	Chiroptera and Carnivora	Infected tissues and fluids, esp. salvia and liquor of CNS			[70,72]
Rota ^Z^	*Group-A-Rotavirus*	Reoviridae	Human and animals	Fecal-oral route			[73]
Venezuelan Equine Encephalitis ^Z,E^	*Alphavirus*	Togaviridae	Rodents		*Culex*		[70,74]
Vesicular Stomatitis ^Z^	*Vesiculovirus*	Rhabdoviridae	Unclear, Grasshoppers (*Orthoptera: Acrididae*) could serve as reservoir	Infected tissues and fluids	Flies *Lutzomyia* and *Simulidae*, Mosquito *Aedes* and Midges *Culcoides*		[75,76]
West Nile Virus ^Z,E^	*Flavivirus*	Flaviviridae	Birds (mainly *Corvidae*)		*Culex*		[77]
Western Equine Encephalitis ^Z,E^	*Alphavirus*	Togaviridae	Wild birds		*Culex* and *Culiseta*		[70,74]
**Bacteria**							
Anaplasmosis ^Z^	*Anaplasma phagocytophilum*		Birds		Ticks (*Ixodes* species)		[78]
Anthrax ^Z^	*Bacillus anthracis*		Spore-contaminated environment	Direct contact		Contaminated objects and premises	[79,80]
Botulism ^Z^	*Clostridium botulinum*		Spore-contaminated environment	Woundinfection		Spore-contaminated food, Inhalation	[81]
Bruccelosis ^Z^	*Brucella abortus and Brucella suis*		Wildboar, Elk	Infected tissues or fluids		Inhalation in overcrowded areas or consumption of raw meat or undercooked animal products	[82,83]
Clostridiosis ^Z^	*Clostridium difficile*		ubiquitous	Direct contact		Spore-contaminated environment incl. Food and airborne	[84]
Glanders ^Z^	*Burgholderia Mallei*		Horses, donkeys and mules	Invasion of abraded or lacerated skin		Inhalation with deep lung deposition	[85,86]
Leptospirosis ^Z^	*Leptospira interrogans*		Rodents	Infected urine and other fluids		Contaminated soil or water	[70,87]
Lyme Borreliose ^Z^	*Borrelia burgdorferi*		Rodents and Birds		Tick (*Ixodes ricinus)*		[88]
Methicillin-Resistant Staphylococcus aureus (MRSA) ^Z,E^	*Strains of Staphylococcus aureus*		Human	Direct contact		Contaminated environment	[89]
Rhodococcus Equi ^Z^	*Rhodococcus equi*		Environmental saprotroph			unclear, probably contaminated environment	[90]
Salmonellosis ^Z^	*Samonella enterica ssp. enterica serovar typhimurium*		Livestock	Fecal-oral route		Foodborne when using infected manure of horses	[91,92]
Streptococcus ^Z^	*Streptococcus equi subsp. zooepidemicus*		Horse	Direct contact			[93]
Tetanus ^Z^	*Clostridium tetani*		Soil or feces of horses and livestock			Contaminated environment	[94]
Tuberculosis ^Z^	*Mycobacteria avium, bovis and turberculosis*					Aerosol and food-borne	[95]
**Parasites**							
Cryptosporidiosis ^Z^	*Cryptosporidium parvum*		Cattle, Horse and pets	Handling infected animal		Food- and waterborne	[96]
Giardiasis ^Z^	*Giardia intestinalis (lamblia)*		Mammals and human	Handling infected animal Fecal oral route		Food- and waterborne	[97,98]
Toxoplasmose ^Z^	*Toxoplasma gondii*		cats			Foodborne by consumption of contaminated horse meat, waterborne	[99]
Trichinellosis ^Z^	*Trichinella*		Rodents, wildboar and domestic swine			Foodborne by consumption of contaminated horse meat	[100,101]
**Fungal infections**							
Dermatophysosis ^Z^	*Microsporum canis* *Micosporum gypseum* *Trichophyton verrucosum* *Trichophyton mentagrophytes* *Trichophyton equinum*		Cats and dogs Soil Bovine Rodents and camels Horses	Grooming, touching		Contaminated objects	[102]
